# Enabling Fieldwork for All (EFFA) Framework: Supporting physical, social, financial, and psychological safety in the field

**DOI:** 10.1126/sciadv.aeb6753

**Published:** 2026-02-27

**Authors:** Lisa L. Walsh

**Affiliations:** AAAS Science and Technology Policy Fellow, the National Science Foundation, Alexandria, VA, USA.

## Abstract

Calls to address fieldwork safety that began in the 1980s have been amplified and expanded in the past decade by the National Academies of Sciences, Engineering, and Medicine and US federal funding agencies. Now, research on fieldwork safety and resulting recommendations are largely siloed by scientific discipline, limiting the spread of data and discussion that could yield rapid change for field research. This review synthesizes literature on fieldwork safety across scientific disciplines, highlighting four facets of safety for leaders and researchers to address: physical, social, financial, and psychological. Literature and real-life events demonstrate that the four facets of safety are interconnected and should be considered together. The review concludes with a synthesis of recommendations for each facet of safety. This review provides principal investigators with accessible, data-driven resources and propose a framework for future research on field safety that will enable cross-disciplinary sharing.

## Teaser

Comprehensive review of fieldwork safety literature across disciplines yields synthesized recommendations.

## INTRODUCTION

A variety of disciplines ranging from biology to political science to archeology uses fieldwork to collect data and advance our understanding of the world. Fieldwork is defined by the US National Science Foundation as research activity that is conducted off-campus or off-site and includes situations ranging from extended excursions in remote locations to brief visits to nearby unaffiliated facilities (*1*). Fieldwork is strongly linked to the identity and culture of some disciplines, including earth science and conservation biology, and early training experiences in the field are often considered to be requisites to pursue a career (*2*–*4*). With immersive learning and emphasis on teamwork, early field experiences serve as recruitment tools while simultaneously increasing trainee self-efficacy and improving graduation rates (*5*).

Conducting fieldwork with research teams drives scientific discovery and can offer unique networking opportunities that enrich career paths. Fieldwork allows individuals to travel to places they might not otherwise have the opportunity to visit, provides novelty and dynamic work experiences, and allows researchers to discover their resiliency and build long-term confidence (*6*). At the same time, traveling to an unfamiliar location with strangers or professional acquaintances carries a variety of risks. Negative fieldwork experiences can lead to reduced productivity, career stalling, costly transfers, and even career abandonment (*7*–*9*).

Since the 1980s, researchers and scientific societies have been calling for increased attention surrounding the safety of research teams going into the field [e.g., (*10*–*17*)]. The type of safety highlighted in these calls varies, as do the parties they challenge to improve or address field safety (e.g., supervisors, institutions, and funding agencies). On the basis of this call-to-action literature (*10*–*17*), along with commentaries and research articles, this review identified four facets of field safety for the scientific community to address: physical, social, psychological, and financial. This paper

1) Provides a literature overview of the available data on safety pertaining to field researchers;

2) Presents the Enabling Fieldwork for All (EFFA) framework to plan, evaluate, and research field campaigns in the context of safety; and

3) Summarizes promising practices published in peer-reviewed journals and white papers as they relate to the proposed framework.

The literature and case studies included in this review represent geoscience, biology, environmental science and conservation, anthropology, sociology, political science, economics, and polar research. Additional disciplines with relevant fieldwork but now missing from the literature include environmental chemistry and toxicology, engineering, history, and agricultural science.

## PHYSICAL SAFETY IN THE FIELD

Here, physical safety for field scientists encompasses minimizing bodily harm ranging from scrapes and sunburns to disease and mortality. Fieldwork is often painted as a feat of endurance and heroism, but this can lead to intense pressure on researchers to “keep up” with their field team and to execute expeditions without any failures, mistakes, or mishaps (*18*, *19*). Researchers in the field face the possibility of injury and illness, and even minor health setbacks can prematurely terminate an expedition depending on the technical skills needed and physical demands of the fieldwork (*20*). Physical hazards reported from fieldwork include mild hazards such as insect bites, sunburns, and blisters, as well as more dangerous hazards including parasitic diseases, local crime, political turmoil, and vehicular accidents (*10*, *21*). In cases of limited field gear availability, women can also face the additional hazard of ill-fitting field clothes due to their body frame, thereby increasing the risk of falls or exposure to extreme weather (*22*, *23*). Access to medical facilities varies widely, with a substantial number of researchers reporting the use of a field site with limited or no access to hospitals or doctors and 67% reporting field sites without refrigeration, a storage condition required for some medicines including insulin (*10*, *21*).

Field scientists have been risking bodily harm for centuries, but the risks have changed over time due to medical and technological advances (*21*, *24*). At least four major hazards identified between the 1980s and 2000s have been ameliorated in the following decades thanks to innovations in pharmaceuticals (e.g., preventative treatments for malaria and hepatitis) and engineering (e.g., portable water purification and fewer vehicles without seat belts) (*10*, *21*, *24*–*26*). For example, airplane and helicopter crashes were the leading cause of death of wildlife workers in the 20th century. Now, with the invention and widespread availability of unmanned aerial vehicles (UAVs), researchers cite these previous mortality cases (*24*) to support the use of UAVs for wildlife monitoring to drastically increase their physical safety [e.g., (*25*, *26*)].

Research on the causes of physical injuries in the field is primarily limited to studies using mortality data. Most deaths in the field were unintentional (e.g., animal attacks, falls, avalanches, drowning; Table 1). The frequency at which vehicular accidents caused deaths indicates the importance of wearing seat belts in vehicles and high-visibility gear for roadside work (*24*, *27*). Four of the 11 students who died were alone when they died, highlighting the danger of solo fieldwork. All intentional deaths reported were due to murder, and workplace death from violence in the field exceeded the average workplace death reported to Occupational Safety and Health Administration. Violence against field researchers was from external actors, rather than coworkers, and was primarily due to contested land rights and political strife, highlighting the importance of understanding the social landscape of a field site (*27*).

**Table 1.** Deaths in the field. Summary of causes of deaths in the field (*24*, *27*). Bold values indicate the most common cause within a publication.

Publication	Sasse (*24*)	Cantine (*27*)
Discipline and years	Wildlife, 1937–2000	Geology, 2000–2021
*N* deaths	91	69
Aviation	**60**	7
Vehicular	6	**14**
Drowning	10	7
Murder	4	13
Disease/medical	1	9
Fall	2	6
Wildlife	1	6
Fire	3	2
Exposure	1	2
Avalanche	1	1
Electrocution	1	1
Heavy machinery	1	1

## SOCIAL SAFETY IN THE FIELD

Here, social safety for field scientists encompasses (i) interpersonal and group dynamics of research teams and (ii) the local social and cultural landscape in and around the field site.

### Research team—sexual misconduct

Most research on social safety has focused on sexual harassment and assault, as academia has the second highest rate of sexual harassment reported for a workplace, and professional and domestic lives are often blurred in the field due to shared living spaces (*28*, *29*). In 2013, anthropologists conducted a survey that galvanized other scientific disciplines to closely examine sexual violence in fieldwork (see Table 2 for a summary of surveys). In their groundbreaking work that surveyed of more than 600 field scientists, Clancy and colleagues (*30*) found that 64% of scientists had personally experienced sexual harassment, 22% had been sexually assaulted, and more than 70% had observed or learned of inappropriate sexual comments made in the field.

**Table 2.** Sexual misconduct in the field. Summary table of published survey results from studies on sexual harassment and assault in the field sciences. Except where noted, responses were specific to witnessing or experiencing sexual misconduct during fieldwork. Overall percentages are provided in bold, and n/a indicate that data or discussion was not provided within the article.

Article	Participants surveyed	Witnessed harassment	Sexually harassed	Sexually assaulted	Trends
Clancy *et al.* (*30*)	Field scientists, primarily anthropologists (*N* = 666)	**> 70%**	**64%**	**22%**	Men targeted by peers
			70% of women	26% of women	Women targeted by superiors
			40% of men	6% of men	
Meyers *et al.* (*8*)	Archaeologists (*N* = 382)	n/a	**68%**	**13%**	Victims typically women early in career
			75% of women	15% of women	
			55% of men	7.5% of men	
Fischer *et al.* (*31*)	Atmospheric scientists (*N* = 265)	**32%**	**27%**	n/a	Victims typically women early in career
			42% of women		
			8% of men		
St. Clair (*34*)	Women in ocean scientists (*N* = 980)	**66%**	78% of women (not field specific)	n/a	Fieldwork was most common location of sexual misconduct, followed by campus
Marín-Spiotta *et al.* (*9*)	Geoscientists (*N* = 2141)	n/a	**14%** within last 12 months (not field-specific)	n/a	Early career participants twice as likely to experience harassment
			20% of women		
			6% of men		
Carlin *et al.* (*23*)	Field based environmental scientists (*N* = 102)	n/a	64% of women	**13%**	n/a
			15% of men		
Perumalswami *et al.* (*33*)	NSF grantees (*N* = 215)	n/a	**53%** (not field specific)	n/a	n/a
			73% of women		
			31% of men		
NSF (*32*)	US Antarctic Program deployers (*N* = 679)	**68%** while deployed	**35%** while deployed	**2.5%** while deployed	Supervisors were less likely to report their victimization compared to nonsupervisors

Sexual harassment experienced during fieldwork ranged from 27 to 68% of survey participants, with up to 75% of women and 55% of men experiencing harassment (*8*, *23*, *30*–*32*). While less frequently evaluated, sexual assault during fieldwork was experienced by 2.5 to 22% of survey participants (*8*, *23*, *30*, *32*). Perpetrators from the local area were reported in rare cases, but most of the harassers were fellow researchers (*30*, *31*). Men primarily reported being targeted by their peers, while women reported being targeted by their superiors (*30*).

Across scientific disciplines, women and trainees were at higher risk to become a victim of sexual misconduct, placing early career women as an especially vulnerable demographic both in the field and on campus (*8*, *9*, *23*, *30*, *31*, *33*). One study that examined the age of participants found that the likelihood of being a target for sexual misconduct during fieldwork peaked between the ages of 26 and 30 (*8*). Being a target of sexual harassment was also more likely, regardless of the setting, for those who identified as disabled or within the LGBTQ (lesbian, gay, bisexual, transgender, and queer) community (*9*).

In multiple studies, most of the respondents were either not aware of a code of conduct or how to report sexual harassment (*30*, *34*). A large portion of respondents indicated that they would not feel comfortable reporting sexual harassment, with supervisors less likely to report being victimized than nonsupervisors. (*32*, *34*). Common reasons to avoid reporting included fear that it would hurt the victim’s career trajectory and concerns that the perpetrator had enough power to affect the investigation (*34*). In addition, respondents largely did not trust management to respond appropriately to their complaint or had already been unsatisfied with the outcome of reporting (*30*, *34*). However, a recent study by the US Antarctic Program found that their strategic interventions to spread awareness were a large success, with 90% of respondents demonstrating that they were familiar with sexual misconduct policies and 85% of respondents indicating that they knew their reporting options (*32*).

### Research team—team dynamics

The general work culture of an institution or department has a strong influence on an individual’s treatment, with more hostile work environments promoting forms of harassment including, but not limited to, sexual harassment, racial harassment, and bullying (*9*, *35*, *36*). Those who have been the target of harassment reported feeling powerless, vulnerable, and excluded from professional opportunities, and many indicated that their work was negatively affected as a result (*7*, *9*, *22*, *36*). While most data on hostile work environments in academia are not fieldwork specific, limited data indicate harassment from academic colleagues increases when off campus (*36*).

Those most likely to be bullied in academia are trainees, while the most commonly reported bully is the academic supervisor (*9*, *35*). Abusive supervision is commonplace in academia, with 84% of individuals reporting being a target at least once (*35*). The most common forms of hostility in academia include encouraging others to mistreat an individual, threatening to erase a position, providing poor recommendations, and removing funding support (*35*). Identity influences both the likelihood of experiencing a hostile academic work environment and the degree of hostility (*35*, *36*). Women were more likely to experience hostility, while international academics experienced more severe levels of hostility (*35*). In both the cases of sexual harassment and academic bullying, most victims chose not to report out of fear of retaliation, and most of those who did come forward were not satisfied with the result (*30*, *34*–*36*). Hierarchical power structures in academia enable harassment (*35*, *36*), and ineffective responses to complaints promote a work environment rife with harassment (*36*).

In an examination of 90 academic institutions, only 40% of fieldwork policy documents included a statement that researchers in the field have a right to safety, and only 5% included a statement that researchers had the right to harassment-free work. Only 10% of risk assessment documents listed discrimination as a potential risk for fieldwork (*37*). Two-thirds of field sites were found to have ambiguous or no rules regarding professional behavior, and when rules were broken, there were typically lenient or no consequences. An absence of clear rules resulted in alienation, gendered divisions of labor, harassment, and assault (*7*). Field sites that had clear rules for professional behavior had specific trainings, meetings, or conversations that articulated the rules and consequences for violations, and expected behavior was modeled by established researchers. In addition, field conditions were designed intentionally to be safe, and staff in charge of a field site were proactive problem-solvers who made reporting mechanisms clear (*7*).

### Local social landscapes

In some communities, the arrival and activity of researchers can lead to gossip and unwanted attention from external parties that interferes with the scientists’ ability to conduct research (*38*). Researchers in international settings may face antiforeigner sentiment and culture shock, yet only 24% of policy documents stated responsibilities specific to international fieldwork (*10*, *23*, *37*). Conditions that are considered most ethical by institutional review boards for human research can clash with the safety of the field researcher, leading the scientists to travel to remote interview locations alone without notifying anyone about where they are going (*39*).

In our review,? data on the field safety of local social landscapes are only available from anthropology (*10*, *21*). Twenty percent of expeditions were reported to have researchers lodging with and living among locals (*10*). When provided with a list of hazards to indicate which they had encountered, 55% of researchers experienced local corruption, more than 26% reported community violence against women or sexual assault, and over 22% had experienced a field site with a high risk of kidnapping or assault (*21*). Thirty-four percent of anthropologists had a member of their team evacuated, 13% had a team member experience violence during fieldwork, and 20% knew of a team member who had been sexually assaulted. When asked if their team had a clear plan for each of the three incidents, respondents were most confident about evacuation plans and least confident about plans responding to acts of violence. Across all three incident types, students were the least aware of existing plans, revealing a gap in trainee preparation for the field (*21*).

## FINANCIAL SAFETY IN THE FIELD

Here, financial safety encompasses (i) personal finances and the associated fears of debt and (ii) limited research funds. Most of the literature on the personal cost of fieldwork focuses on students and trainees. Many students already face exorbitant costs directly related to their education, and the financial burden of field apparel and gear can deter students from pursuing field-related courses, internships, and ultimately careers (*40*). In 2022, estimates for outdoor gear ranged from $600 to $2185 USD (*41*). As students progress in their research, they may require additional expensive gear, training, and documents, such as SCUBA (self-contained underwater breathing apparatus) gear, certifications, and a passport. Socioeconomic background was reported to be a barrier to fieldwork participation by 50.5% of field scientists (*23*).

Popular disciplines such as conservation biology and wildlife research often rely on unpaid work to keep research costs low, touting that the experiences gained from unpaid work will allow trainees to get their foot in the door of a highly competitive discipline. For example, in 4 months of job postings on two wildlife websites, 38% of postings were for unpaid or “pay to work” positions (*2*). Four years later, 90% of primatology fieldwork positions posted were unpaid, with 65% of positions indicating that no expenses, including lodging, would be covered (*42*). Long, unpaid or underpaid field campaigns are an especially strong financial deterrent, as the time away from home may conflict with work requirements or family caring needs that take priority over field experience (*23*, *40*). For example, 37% of women who had participated in Antarctic research indicated that they spent at least 20 hours a week providing care to a family member. Therefore, long stints of work at a field station or remote fieldwork strained their familial relationships and conflicted with family planning. Multiple women stated that they could not return to Antarctic fieldwork after having a child due to caretaking requirements (*22*).

For trainees seeking the requisite experience to pursue field science professionally, they can attend a field course rather than pursue an unpaid or underpaid field technician position. As of 2020, these courses ran for 4 weeks and cost between $2100 and $9200 USD before airfare and field gear (*42*, *43*). In 4 weeks, attending a field course rather than working could amount to thousands of dollars in lost wages (*43*). Trainees interested in learning about field safety often must pay out of pocket for training (*44*).

The budget available for a field expedition affects the size of the field team, materials provided to the team, and time allotted for fieldwork. As the cost for travel and lodging increases, the size of the research team often shrinks (*10*). Fieldwork days can be cancelled due to inclement weather conditions, reducing productivity, especially in circumstances when the time window of fieldwork cannot be extended due to budgets or travel itineraries (*45*). Depending on the field camp or station where the team is staying, these unproductive field days can be quite costly. In an extreme example, as of 2020, Arctic camps can cost upward of $6000 USD a day per team member (*46*). Financial pressure can lead to compromising physical safety by encouraging researchers to work in hazardous environmental conditions to maximize field days or work alone to keep labor costs low.

## PSYCHOLOGICAL SAFETY IN THE FIELD

Here, psychological safety for field scientists encompasses the psychiatric wellness of field researchers, including (i) managing preexisting conditions when in the field and (ii) preventing scenarios that are known to promote mental health issues including stress and anxiety. On the basis of research on mental health in academia, a substantial portion of field researchers has experienced a mental health issue in their day-to-day life (*47*–*49*). Graduate students are at a heightened risk for depression, anxiety, substance abuse, and emerging mood disorders (*47*, *48*), and up to 60% of faculty meet criteria for anxiety and depression (*4*, *49*). Once in the field, preexisting mental health issues can be exacerbated by a variety of factors. Physical exhaustion from long work hours (e.g., 8 a.m. to 11 p.m.), lack of sleep, isolation, and close quarters, along with the social pressure to drink and socialize whenever the research team had down time, were all reported as conditions that worsened the psychological health of individuals with preexisting conditions (*4*).

Regardless of preexisting conditions, fieldwork has been reported to negatively affect between 5 and 18% of researchers’ mental health (*21*, *50*). Mental health issues reported from fieldwork include culture shock, anxiety, mood disorders, repatriation stress, hallucinations, sleep disorders, personality disorders, and substance-related disorders (*10*, *50*). In addition to those listed in the previous paragraph, other conditions unique to fieldwork can also affect mental health. For example, many field sites have no toileting option, and not knowing where to relieve oneself or how to deal with menstruation in the field can cause distress for researchers, especially in open areas without vegetative cover (*10*, *21*, *22*, *40*). In the event of an emergency in the field, such as a fall or wildlife attack, adrenaline causes cortical inhibition, limiting a researcher’s access to higher brain functions including decision-making and memory recall which can result in slow reaction times, compromising physical and social safety and increasing anxiety regarding work at the associated field site (*51*).

Psychological safety in the field is most vulnerable in sites of isolation, such as vessels and remote camps or stations. Particular attention has been paid to polar-based researchers because of the long history of psychological events reported, extreme elements, and heightened levels of isolation experienced. In a comprehensive review of the literature, the most common symptoms reported for polar expeditions included impaired sleep and cognition, depressed affect, and increased interpersonal conflict (*6*). After originally passing their psychiatric screening to embark on polar research, ~5% of polar researchers had symptoms meeting Diagnostic and Statistical Manual of Mental Disorders (DSM-IV) criteria for a psychiatric disorder at the end of their field campaign. The most common DSM-IV diagnoses for polar researchers included mood disorders, adjustment disorders, and sleep disorders (*50*).

## FRAMEWORK TO PROMOTE HOLISTIC FIELD SAFETY

When researching or discussing fieldwork safety, the four facets of safety outlined above tend to be siloed from one another. For example, experts in occupational health and safety or wilderness first aid often focus on physical safety [e.g., (*52*)], while campaigns in remote areas might focus on psychological safety due to isolation [e.g., (*50*)]. The research emerging on sexual misconduct in the field has also produced discussions that focus on the social safety of a field team [e.g., (*15*, *53*)]. In an effort to drive the community to consider a more holistic approach to field scientist safety, Nordseth and colleagues (*54*) proposed the Fieldwork Wellness Framework composed of eight dimensions of wellness: physical, emotional, intellectual, social, environmental, financial, occupational, and spiritual. By distilling these eight dimensions down to the four facets of safety presented in this paper (physical, social, financial, and psychological), the interconnections between the types of safety become clear.

For example, the financial costs of fieldwork in South America and Africa compared to North America and Europe drove more North American anthropologists to conduct solo fieldwork, compromising their physical and social safety (*10*). Financially limited researchers and teams may not have the equipment and gear necessary to keep the team safe from the elements and environments they face. Team members from precarious socioeconomic backgrounds reported taking more risks and refusing to report issues out of concern for losing their job (*23*, *36*). As highlighted by polar researchers, physical discomfort can increase stress and interpersonal conflict (*6*). Poor social safety, especially sexual assault or harassment, has been cited as a factor that negatively affects researcher mental health (*7*, *22*, *23*).

### Enabling Fieldwork for All Framework

**Fig. 1.**
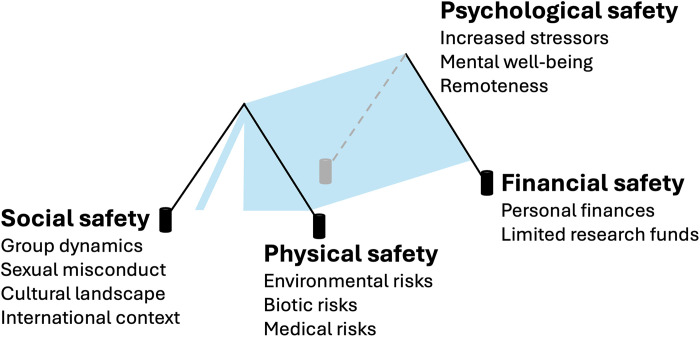
EFFA Framework. Visualization of the EFFA Framework. Each facet of safety is depicted as a tent stake to symbolize that removing one can comprise others, thereby collapsing overall safety. Aspects to consider are listed below each facet.

Building off the Fieldwork Wellness Framework (*54*), the literature reviewed above, and the interconnections between facets of safety, the author proposes a simplified framework of four facets of safety required to enable safety for a field campaign: the EFFA Framework. Each facet of safety (physical, social, financial, and psychological) is illustrated as a tent’s stake symbolizing the requirement for all four to support a researcher’s overall safety (Fig. 1). If one stake is weakened, then it can compromise other stakes and the entire tent collapses. Ultimately, the EFFA Framework was created to underline the importance of addressing all four facets of safety when discussing and assessing field safety.

**Fig. 2.**
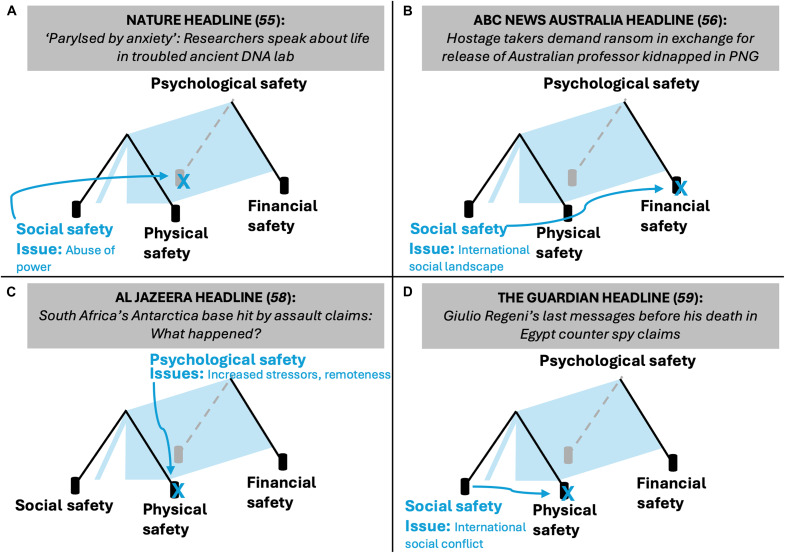
Field safety case studies from the news. Visualization of news headlines mapped onto the EFFA Framework. (**A**) A social safety issue compromises psychological safety, (**B**) a social safety issue compromises financial safety, (**C**) a psychological safety issue compromises physical safety of team, and (**D**) a social safety issue compromises physical safety (*55*, *56*, *58*, *59*).

Real events highlighted in the news demonstrate EFFA’s foundation: the weakening or loss of one safety facet compromises others. For example, after a university investigation, a well-established scientist was suspended for bullying trainees in his laboratory. The investigation revealed multiple instances of verbal abuse and a wide-spread fear of retaliation among laboratory members. This toxic laboratory culture led to students developing stress-related health issues (Fig. 2A) (*55*). A team of Australian and Papua New Guinean anthropologists conducting fieldwork in a remote part of Papua New Guinea (PNG) were kidnapped by an armed criminal group and held for ransom. PNG government officials noted that this was part of an emerging criminal trend in the area (*56*). In addition to the physical and psychological threats of being kidnapped, this dynamic social landscape compromised the financial well-being of the researchers and their institutions (Fig. 2B). The kidnapped researchers were released after 1 week of captivity (*57*).

In a nine-person team deployed to a remote South African base in Antarctica, a staff member began to act erratically and became increasingly agitated. His behavior escalated, and he physically assaulted the team lead and made death threats to other team members (*58*). The increased psychological stressors facing polar researchers led to a physical safety issue (Fig. 2C). The most serious and distressing case studies result in the loss of life of researchers. A political economy graduate student conducting research on trade unions in Egypt became fearful that he was being surveilled. The student was later kidnapped under wrongful suspicion of being a spy, tortured, and killed (*59*). Political turmoil and mistrust in foreign researchers contributed to the dissolution of physical safety in the field (Fig. 2D).

## PROMISING PRACTICES

Under the EFFA Framework, the physical, social, psychological, and financial safety of team members should all be considered for any field campaign. Below, the author summarizes peer-reviewed resources for overall safety, as well as promising practices for each of the four EFFA facets of safety (compiled in Table 3). Promising practices are included for field researchers, team leads and supervisors, departments and institutions, and government agencies, as no single party should be held solely responsible for preparing the field team for a safety expedition. Each section of promising practices is organized chronologically, from pre-expedition planning to post-expedition follow-ups.

**Table 3.** Promising practices for field safety. Summary of promising practices presented for the four EFFA Framework facets of field safety. Parties that can implement each practice are listed within parentheses. G, government agencies; I, institutions; P, PIs; T, team members.

Practice	Summary
Physical	Foster a positive work environment that promotes sharing concerns and allows members to decline activities with which they are not comfortable (P).
	Plan together, add redundancy to team knowledge, pack intentionally (P, T).
	Use the buddy system, check- ins, and briefings for fieldwork (P, T).
	Enable adequate sleep by restricting work hours, reducing alcohol consumption (P, T).
	Foster road and vehicular safety with hi- visibility vests, multiple drivers (I, P).
	In cases of remote work, budget for medical evaluation and evacuation insurance (I, P).
Social	**General**
	Team develops a code of conduct that is iteratively updated (P, T).
	Code of conduct is shared with all participating institutions and should be discussed by all participants before fieldwork (I, P, T).
	**Local social landscapes**
	Plan as a group on how to mitigate encounters with external parties, articulate exit triggers (P, T).
	Avoid use of dating apps in fieldwork locations (P, T).
	Purchase apparel or vehicle decals that identify the team as affiliated researchers and alert locals ahead of time to research plans (I, P).
	Include local laws, attitudes, and norms in the code of conduct (I, P, T).
	Schedule check- ins with a clear action plan if a check- in is missed (I, P, T).
	**Team dynamics**
	Lead by example with respect; provide everyone with learning opportunities in the field (P).
	Use team input on lodging arrangements; equally distribute domestic chores across team (P, T).
	Provide clear communication about sanitary needs and toileting provisions (P, T).
	Diffuse power by having multiple leaders and secondary mentors in the field (P, T).
	Evaluate working conditions throughout the field campaign to make improvements (I, P).
	**Sexual misconduct**
	Address minor issues before behavior escalates (P, T).
	Articulate what appropriate behavior is, how to report misconduct, and post reminders (I, P).
	Provide multiple reporting options, including nonmandatory reporters and 3rd party reporters (I, P).
	Conduct team exercises before fieldwork to run scenario- based activities to improve awareness (I, P).
	Provide interactive bystander intervention training to empower team (I, P).
	Institutional policies are clear and enforced (I).
	Institution provides support through funds and trauma- informed workshops (I).
	Establish cross- institutional reporting policies to end “pass the harasser” (I).
	Share misconduct reports with all institutions and funding agencies involved (I).
	Funding agency should have requirements for reporting misconduct, submitting safety plans (G).
Financial	**Personal finance**
	Post transparent recruitment communications that indicate level of support (I, P).
	Offer field methods courses and field trips within courses to allow students to use traditional financial aid to access field training (I).
	**Research budgets**
	Cite safety literature in budget justification to provide data- driven argument (P).
	Prioritize safety over data collection, especially in hazardous conditions and time- limited field seasons (P, T).
	Survey the department to create a shared repository for field gear and equipment to reduce the need for new purchases (I, P).
	Factor safety expenses into your budget including gear, trainings, personnel for paired work (I, P).
Psychological	Plan ahead, discuss stressors, and personalize support plans.
	Ensure everyone knows crises contacts and has devices for communication.
	Promote trust in the team; leaders demonstrate vulnerability.
	Obtain resources for telehealth; debrief and follow- up after field campaign.
	Provide Mental Health First Aid training.
	Reduce stressors like no downtime, drug use.

When preparing for safe research, what constitutes the “field” should be expanded beyond remote, rugged landscapes, as leaving the infrastructure and resources readily available on campus deserves attention to safety and well-being no matter the location (*60*). The same attention and resources provided to laboratory safety protocols should be applied to fieldwork, and trainees with limited field experience deserve a level of support that allows them to conduct safe, successful fieldwork (*45*). Guidance and training on how to lead safe field campaigns will improve both the scientific research and the professional growth of scientists (*29*, *44*). Anonymous surveys can be leveraged to better understand the needs and concerns of researchers before fieldwork to tailor safety plans and training (*61*). In one forestry department, it is a requirement for each graduate student to develop a safety plan for advisor approval and submit it to the department chair before they can conduct fieldwork. This signals to students that proactively addressing safety is equally as important as their research plan (*45*). When surveyed, scientists were enthusiastic about training for field medicine, preventing sexual assault, and addressing mental health in the field. They were especially enthusiastic about receiving assistance to remove or reduce financial barriers to pretrip clinics (*21*).

A clear communication schedule should be developed by the team before departure, including steps the check-in person will take when a check-in is missed (*45*). Emergency contacts and medical information should be prepared in the event of an emergency for all involved (*44*). There are multiple published resources available to help guide field safety planning, including a template safe fieldwork plan (*62*). Rudzki and colleagues (*61*) provide a long list of resource articles, as well as recommendations for writing a field safety manual. They recommend including researchers with a variety of backgrounds and experiences to enrich the conversations and development of a safety plan. Daniles and Lavallee (*45*) provide a general field safety framework to develop a safety plan and an associated questionnaire to help researchers identify hazards that should be mitigated in their plans (*45*). A checklist for principal investigators (PIs) and supervisors to use before, during, and after field research can be used to make sure codes of conduct and other policies exist or are written, prompt open two-way communication, conduct regular check-ins, and find relevant training (*53*).

During fieldwork, whenever possible, researchers should conduct fieldwork in groups using the buddy system. In the event that a member of the field team is a target of harassment, it is recommended that the victim is provided with a variety of reporting options that are not limited to mandatory, formal reports (*63*). While mandatory reporting policies may have been implemented with the intent to increase transparency and better address sexual assault, it often creates barriers for survivors to seek assistance after an assault. Mandatory reporting strips the survivor of anonymity, removes autonomy from the survivor, and compromises mentor-mentee relationships (*64*). Institutions and team leads should recognize that the fear of retaliation after coming forward is a persistence problem and be proactive in providing support to victims (*63*).

In the long term, funders, institutions, and principal investigators should view the safety and well-being of a research team as a fundamental aspect of ethical research (*11*, *29*, *65*). For institutions, the National Academies (NASEM) recommends data-driven approaches, which can include climate survey data at the laboratory, department, and institutional level. By routinely surveying researchers, issues that need to be addressed can be identified, and policies can be evaluated and iteratively improved. When policies have a longitudinal record of supporting researchers, they can be institutionalized for lasting effects. In terms of effective policies, NASEM recommends educational programs that help foster a supportive, harassment-free work environment and policies that clearly articulate behavioral expectations and consequences for violations. It is also important that institutions create spaces for mentors and mentees to be informed on how identities can influence someone’s experience in the field (*63*).

Efforts to improve field safety for the department or institution should be recognized by academic leadership and scientific societies. This can include awards, an area to feature these efforts in promotion packages, and compensating faculty for their time by formalizing a field safety committee to recognize the time they put into advancing field safety (*63*).

Government agencies can take action to promote transparency and accountability at the individual researcher level and at the institutional level. Agencies can require institutions to routinely collect and report out on data collected from field researchers. For example, the US CHIPS and Science Act of 2022 requires the US National Science Foundation to collect data on sexual and gender harassment (*66*). Federal agencies also have the power to hold institutions accountable and withhold research funds from noncompliant researchers or institutions (*63*).

Government agencies and research institutions can work to fill gaps in knowledge to improve the research enterprise’s ability to protect field researchers (*63*). The US CHIPS and Science Act of 2022 mandated the US National Science Foundation to provide research awards to better understand the factors driving sexual harassment and develop and evaluate interventions (*66*). As revealed by the literature review, less research attention has been paid to social landscapes, financial safety, and psychological safety in the field.

### Promising practices—physical safety

Researchers who conduct physically and technically challenging fieldwork emphasize the importance of planning and having enough training to respond to dynamic or volatile conditions (*20*). According to occupational health and safety compliance experts, creating workplace safety plans for environments encountered during fieldwork requires special attention to the unique challenges of the environment and the variation of work tasks involved (*52*). Outlining the specific activities and environments for fieldwork can be used to determine potential hazards and how to address them (*52*). Tailoring a safety plan to the field campaign also requires all involved to actively participate, even those who are well-versed in traditional field safety (*20*, *29*). The finalized safety plan outlining how to address hazards serves simultaneously as a training guide, manual, and contract between the team members (*52*).

Eifling (*51*) provides resources to run a 2-hour training that enables a team to strategize about field safety. The training includes a SWOT (strengths, weaknesses, opportunities, and threats) analysis, risk analysis, and activities to strategize ways to mitigate and respond to hazards that ensures multiple team members know how to use the relevant safety items and equipment (*51*). A medical evaluation before the field season is recommended when hospitals are inaccessible to reduce the chance of medical emergencies that would go untreated or require evacuation (*52*). In addition, funds should be allocated for an emergency evacuation of the team (*67*).

During the field campaign, it should be made transparent who is responsible for determining stop-work events and the conditions that would trigger such a decision. Daily briefings that identify collection sites and discuss “near misses” can be used to keep the team informed and iteratively improve preparation for physical safety (*31*, *52*). The research team should feel comfortable sharing concerns with each other at any time, highlighting the importance of trust within the team, and it should be made clear to team members that they can decline participating in a hazardous activity without repercussion (*20*, *52*). Accidents are more likely to happen when maximizing work output takes priority over safety, and experienced researchers stress how important getting enough sleep is to have a safe field campaign (*20*, *52*). Data from fieldwork deaths suggest that special attention to road and vehicular safety is warranted, including the use of high visibility gear and seat belts (*27*).

In some disciplines, the emphasis on physically strenuous field experiences for students was driven by industries and careers that are now evolving or being phased out as technologies improve. It is critical that academia evolve alongside industry to provide trainees with up-to-date career training rather than continuing to require a certain number of hours in the field [e.g., UAV training and remote sensing; (*23*, *40*)].

### Promising practices—social safety

To empower team members, it is recommended that leaders ensure everyone is aware of policies and underline the importance of social safety. A field team should work together to develop a code of conduct that acts as a living document articulating how the team members are expected to engage with each other, with external parties, and with the community in which they are conducting research. This code of conduct should be returned to routinely by the team to discuss and update accordingly (*44*).

#### Sexual misconduct

Sexual harassment and assault in the field should be viewed as health and safety issues as well as research integrity issues (*29*, *31*, *68*). While a variety of organizations and field stations have well-established approaches to physical safety in the field (e.g., wildlife and plant awareness trainings, first aid trainings, and gun safety), a similarly proactive approach should be embraced to reduce sexual harassment and assault (*69*). Here, the author breaaks down the recommendations into three categories: preparation, policy, and reporting.

*Preparation*. A precampaign list of items to review including the code of conduct, mandatory and recommended trainings, and substance use policies can be leveraged to ensure that everyone involved in fieldwork is familiar with behavioral expectations and social policies (*29*). Conducting a community readiness evaluation, running a scenario-based risk activity, and group safety trainings can all help the team prepare for the field and identify gaps in their preparation. Group training activities also foster team bonding and improve communication to a degree that watching a safety training video does not (*69*). Leaders should be mindful to avoid training burnout for their team, and the team should be compensated for their time during trainings (*29*).

An especially effective form of training is bystander intervention, which has been found to improve self-efficacy and promote action against sexual misconduct (*28*, *29*, *31*, *68*, *70*, *71*). Bystander intervention training empowered multiple male colleagues to intervene in cases of harassment while out in the field (*31*). A 90-min bystander intervention workshop that included knowledge-based interventions, social modeling, and mastery experiences yielded a statistically significant, long-term increase in self-reported knowledge and confidence. Individuals who received training had a higher intent to encourage others to act, and training was impactful across career stages, indicating that even brief trainings can help address sexual misconduct in the field (*72*).

*Policy*. There are many instances of harassment that do not rise to the level of a Title IX Office investigation (or equivalent) but still contribute to a toxic, unsafe working environment (*29*). When supervisors and team leaders address issues early, they can help reduce the incidents that meet Title IX requirements. By clearly sharing with the team what are inappropriate behaviors and verbal remarks, how to report incidents, and consequences for misconduct, field leaders reduce the fear of reporting, discourage harmful behavior, and provide structural support for staff responding to a complaint (*29*, *34*). Participants can be reminded of how to report by posting information in private spaces and providing electronic copies on everyone’s devices (*29*).

The strongest predictor of sexual misconduct is an organization’s climate surrounding the tolerance of inappropriate behavior (*28*). To address this, institutions should have clear policies and be transparent in how misconduct is handled (*68*). An accessible sexual harassment policy that is directly discussed signals to all participants that inappropriate behavior is not tolerated (*34*). This can be further underlined by including sexual misconduct policies within a code of conduct or community agreement that each team member signs before the field season (*69*). Institutions can demonstrate their continued support by providing administrative and financial backing to the policies, such as purchasing communication devices, privacy screens, and hosting workshops including field safety and bystander intervention workshops (*29*). Too often, institutions do not communicate effectively with each other and “pass the harasser.” Establishing cross-institutional communication policies surrounding misconduct can help prevent this phenomenon (*29*).

Sexual misconduct is detrimental to the productivity of a research campaign and the scientific pursuits of all involved. Funding agencies should have policies for responding to such incidents in the field, as it harms their financial and professional involvement (*29*). In October 2018, the National Science Foundation published Article X in the Federal Register, providing requirements for institutional reporting as well as consequences for grants belonging to researchers who commit harassment. This demonstrates how funding can be leveraged as a way to exact change (*34*). An additional lever to exact change is the requirement of safety plans by funders before awarding grants involving fieldwork (*29*).

*Reporting*. There should be multiple, clear reporting options for victims, including nonmandatory reporters (e.g., non–Title IX Office options). Anyone identified as someone to report misconduct to should receive routine trauma-informed training and be familiar with the resources available to survivors (*29*, *64*). If it is not feasible to have dedicated staff on site to respond to reports, then an off-site staff member who can be reached by communication device at all hours should be made available. Reports of misconduct should be shared with all institutions involved and relevant funding agencies. Field members should be able to contact any institution involved, and a third party option for reporting should also be made available to mitigate conflicts of interest (*29*). In cases where reporting sexual assault is mandated by law (e.g., Title IX), it is recommended that institutions limit the number of mandatory reporters to academic leaders (e.g., deans and department chairs) and clearly communicate this list across the institution to mitigate the problems caused by mandatory reporting. Providing survivors with confidential options and support systems allow them to maintain autonomy and anonymity while still receiving vital support (*64*).

#### Team dynamics

The tone of a field campaign is established by leadership, so they should clearly articulate expectations and responsibilities, demonstrate respect to everyone, follow through on enacting repercussions for those who act inappropriately, and support individuals who come forward to report misconduct (*37*, *68*). Ensuring that all team members feel valued, heard, and included will reduce isolation of newer or less experienced team members (*44*). For example, field site leadership worked to enable a student with a physical disability and a student with an injury to fully contribute to field efforts (*7*). Field leaders should be trained in conflict resolution to further manage team dynamics. When overnight stays are involved, everyone should have a choice in their lodging arrangement, and the distribution of labor involving both housekeeping and food preparation should be distributed evenly across the team (*44*, *45*). Clear communication about toilet breaks, sanitary needs, and privacy during fieldwork should also be standard (*23*, *40*, *44*).

It is recommended that leaders or departments evaluate working conditions before, during, and after a field campaign (*40*, *44*). Surveying participants to gauge the working climate and culture can provide space for team members to offer recommendations and allow field leaders to adjust policies and protocols and address arising issues before they result in substantial problems that impair research productivity (*29*, *53*, *63*, *68*).

While leadership is instrumental in establishing and maintaining a positive work environment, such responsibilities should be distributed across multiple individuals to reduce power imbalances between student and advisor or staff and supervisor (*34*). Diffusing power across multiple individuals provides team members with nonchain of command reporting options can mitigate the fear of retaliation, improve morale, and reduce the risks of sexual misconduct (*29*, *34*). If bringing additional leaders into the field is impractical, then the field leader can instead provide the team with contact information for the field leader’s supervisor (*68*).

#### Local social landscapes

Fieldwork exposes researchers to cultures different from their own to varying degrees. This can include interacting with other languages, ways of life, perspectives on natural resources, laws and behavioral norms, and inappropriate gestures. Being aware of how to respectfully act in the cultural landscape of the fieldwork can improve both the safety of researchers and the relationship between locals and the visiting researchers (*44*). Field safety plans presented before the field campaign should include information about local attitudes and laws, and how to interact with locals should be written into the team’s code of conduct. Worst-case scenarios should be identified ahead of time, and work stop and evacuation thresholds (e.g., political unrest and stalking) clearly outlined to prioritize safety over research (*73*).

Demery and Pipkin (*73*) argue that it is the responsibility of the supervisor, field team, and department to share in preparing researchers for scenarios they might face in the field. The supervisor should work alongside the researcher to formulate strategies and protocols for mitigating risk due to the local social landscape. How a researcher is received by locals can be influenced by identity, including both protected (e.g., disability, gender, and race) and unprotected characteristics [e.g., economic class and weight; (*73*)]. Yet, many fieldwork policy documents failed to mention how a researcher’s identity can influence their risk in the field (*37*). The supervisor or field policy document should inform the team about demographic characteristics that might be at heightened risk for a given field site and help them prepare for such risks (*37*, *39*, *73*). This will require that the supervisor or their institution do investigation ahead of time to assess the social landscape of their field site. Violence against field geologists from external actors underlines the importance of knowing who else uses the land, which may include knowing hunting seasons and alerting local game wardens to research activity (*27*). Mitigation strategies also include purchasing institutional apparel for all field researchers and using institutional car magnets to help the researchers establish credentials. Allocating funds to print and distribute informational postcards to field site neighbors to let them know who will be in the field can further ameliorate issues with the community (*73*).

During the field campaign, dating apps and social media can lead to stalking or luring and violence, so careful consideration should be used before using such geolocating apps (*74*). In cases where the researcher is traveling alone or conducting confidential fieldwork, recommendations include scheduling a check-in call, providing a sealed itinerary to open if a scheduled check-in is missed, and brainstorming how to proactively address harassment from external parties (*39*).

### Promising practices—financial safety

Recruitment communications for student field opportunities should be transparent about stipends, support services like housing and fee waivers, and the schedule of any funds disbursed (*75*). Local field trips and university field method courses allow students to use financial aid to gain important experience while minimizing out-of-pocket costs (*43*). Society-organized and funded field opportunities, funding agency support, and institutional support via donors can all increase the opportunities for trainees to experience fieldwork (*42*).

A field gear closet is one way that departments can reduce the financial barrier of fieldwork for students (*40*). Building from this idea, Devitz (*76*) proposed the Equipment Repositories for Accessibility (ERA) model to improve accommodations infrastructure for field researchers. Under the ERA model, an institution or department anonymously surveys their researchers to identify needs, leverages equipment grant funds to purchase items for accommodation, and shares equipment across researchers like a library. This puts the financial burden and responsibility of maintaining equipment on the institution, rather than on individual researchers (*76*).

Lack of financial compensation serves as a notable barrier to fieldwork for those without independent wealth. Field technicians should be paid, and their salaries should be budgeted in grant applications (*2*). Research projects that consider safety throughout the planning phase can craft their budget to promote the well-being of their research team. As highlighted in the literature review section, there is a growing body of peer-reviewed articles available for researchers to cite in budget justifications regarding the safety of their research team [e.g., (*4*, *10*, *21*, *27*, *30*)]. As recommended in the physical safety section, institutions and field leaders should cultivate a working environment that prioritizes safety over data collection while in the field—a day without fieldwork is better than a week of fieldwork lost due to accident or injury (*52*).

### Promising practices—psychological safety

Team members’ experience level, employment status, physical ability, and other traits can affect how they feel both physically and mentally during fieldwork (*44*). Before a field campaign, a health and safety meeting should be conducted that provides information about the field setting, work expectations, predicted hazards, and a packing list to enable the team to proactively identify gaps in resources and abilities. This planning stage includes recognizing the cognitive impact that a change in circadian rhythm may have on team members and making sure that all members have the appropriate time and resources to deal with hygiene needs in the field (*44*, *52*). Mental Health First Aid training can provide field directors and team members with the tools necessary to identify and address researchers in psychological distress (*67*).

Promoting a work culture in which team members feel safe voluntarily disclosing their mental health issues or concerns is a critical step in promoting psychological safety, and it enables the team to create support and communication plans (*67*). One way a leader can reduce stigma around mental health and increase team trust is by sharing a story of when they needed help. If the team members trust each other, then they can choose to disclose stressors with one another and discuss plans to mitigate these stressors. Together with the field director, each team member should produce a support plan that identifies resources they will need such as cell service, satellite phones, or even evacuation insurance. For researchers with existing or recurring mental health conditions, they should consult with their care team before fieldwork to establish a support plan (*67*). Institutions and field camps should consider establishing standards for social situations known to deteriorate mental health [e.g., excessive alcohol use, drug use, and no downtime; (*4*)].

During the field campaign, leaders should foster a positive, affirming work environment with a zero-tolerance policy for inappropriate behavior (*4*, *14*). Strong leadership, shared common goals, and transparent work strategies help reduce interpersonal work stressors (*6*). For example, when team members show signs of physical distress, more experienced members need to take the signs seriously, even if they themselves are comfortable. All team members should know how to contact crises hotlines, institutional counseling services, and victim advocates. Team leaders should demonstrate self-care and schedule downtime for the team (*44*). In cases of remote, isolated fieldwork, access to psychiatric telehealth consultations and other support systems, as well as debriefing with clinicians in-person after the field campaign, can help mitigate mental health issues during and after the field season (*6*, *67*).

## SUMMARY AND OUTLOOK

As highlighted by Eifling and Klehm (*21*), risks change over time thanks to advances. The EFFA Framework can be used to identify risks that demand increased attention to generate innovations in field safety. Datasets on field safety are piecemeal and typically discipline specific, with anthropology now representing the most prolific discipline in peer-reviewed literature. Data missing include information on local social safety for researchers and how limited research budgets affect the safety of teams. In cases where multiple disciplines examine the same facet of safety, the data available are not consistent across projects (Table 2). Creating a validated survey instrument based on the EFFA Framework that can be used across disciplines would enable a more cohesive understanding of the current landscape of field safety.

The EFFA Framework can serve as a foundation to help field teams plan, station leaders and principal investigators evaluate, and researchers identify areas requiring increased attention to improve conditions for field scientists. One such area of attention would include collecting empirical data to more closely evaluate the relationships between the four facets of safety. Given the administrative burden already imposed on scientific investigators, the EFFA Framework offers a streamlined, easy-to-implement framework with just four areas of focus. This framework can equip principal investigators and scientific leaders with the background summaries necessary to argue for funding and departmental attention to holistic field safety.

## References

[1] National Science Foundation (NSF), “Proposal and Award Policies and Procedures Guide,” Chapter II.D.2. (National Science Foundation, 2023; https://nsf-gov-resources.nsf.gov/2022-10/nsf23_1.pdf?VersionId=VQHMy1XFClNhULRMabdaeCqYvbgykldV).

[2] A. M. V. Fournier, A. L. Bond, Volunteer field technicians are bad for wildlife ecology. Wildl. Soc. Bull. 39, 819–821 (2015).

[3] A. Stokes, A. D. Feig, C. L. Atchison, B. Gilley, Making geoscience fieldwork inclusive and accessible for students with disabilities. Geosphere 15, 1809–1825 (2019).

[4] F. Tucker, J. Horton, “The show must go on!” Fieldwork, mental health and wellbeing in Geography, Earth and Environmental Sciences. Area 51, 84–93 (2019).

[5] R. S. Beltran, E. Marnocha, A. Race, D. A. Croll, G. H. Dayton, E. S. Zavaleta, Field courses narrow demographic achievement gaps in ecology and evolutionary biology. Ecol. Evol. 10, 5184–5196 (2020). 32607142 10.1002/ece3.6300 PMC7319162

[6] L. A. Palinkas, P. Suedfeld, Psychological effects of polar expeditions. Lancet 371, 153–163 (2008). 17655924 10.1016/S0140-6736(07)61056-3

[7] R. G. Nelson, J. N. Rutherford, K. Hinde, K. B. H. Clancy, Signaling safety: Characterizing fieldwork experiences and their implications for career trajectories. Am. Anthropol. 119, 710–722 (2017). 10.1111/aman.12929 PMC13042574 41929848

[8] M. S. Meyers, E. T. Horton, E. A. Boudreaux, S. B. Carmody, A. P. Wright, V. G. Dekle, The context and consequences of sexual harassment in southeastern archaeology. Adv. Archaeol. Pract. 6, 275–287 (2018).

[9] E. Marín-Spiotta, E. J. Diaz-Vallejo, R. T. Barnes, A. Mattheis, B. Schneider, A. A. Berhe, M. G. Hastings, B. M. Williams, V. Magley, Exclusionary behaviors reinforce historical biases and contribute to loss of talent in the earth sciences. Earth’s Futures 11, e2022EF002912 (2023).

[10] N. Howell, Health and safety in the fieldwork of North American anthropologists. Curr. Anthropol. 29, 780–787 (1988).

[11] S. Bahn, Keeping academic field researchers safe: Ethical safeguards. J. Acad. Ethics 10, 83–91 (2012).

[12] K. St. John, E. Riggs, D. Mogk, Sexual harassment in the sciences: A call to geoscience faculty and researchers to respond. J. Geosci. Educ. 64, 255–257 (2016).

[13] M. A. Rinkus, J. R. Kelly, W. Wright, L. Medina, T. Dobson, Gendered considerations for safety in conservation fieldwork. Soc. Nat. Resour. 31, 1419–1426 (2017).

[14] C. M. John, S. B. Khan, Mental health in the field. Nat. Geosci. 11, 618–620 (2018).

[15] K. B. H. Clancy, L. M. Cortina, A. R. Kirkland, Use science to stop sexual harassment in higher education. Proc. Natl. Acad. Sci. U.S.A. 117, 22614–22618 (2020). 32817430 10.1073/pnas.2016164117 PMC7502731

[16] D. J. Amon, Z. Filander, L. Harris, H. Harden-Davies, Safe working environments are key to improving inclusion in open-ocean, deep-ocean, and high-seas science. Mar. Policy 137, 104947 (2022).

[17] B. W. Blonder, Carrying the moral burden of safe fieldwork. Bull. Ecol. Soc. Am. 104, e02031 (2022).

[18] G. Rose, *Feminism and Geography: The Limits of Geographical Knowledge* (Polity, 1993).

[19] S. Maguire, Gender differences in attitudes to undergraduate fieldwork. Area 30, 207–214 (1998).

[20] S. Jamal, Fieldwork skills in the extreme. Nature 631, 695–697 (2024). 39009748 10.1038/d41586-024-02311-x

[21] K. P. Eifling, C. E. Klehm, CAMPS: Combined Anthropology Medical Preparation Survey, 2018. Curr. Anthropol. 61, 798–807 (2020).

[22] M. Nash, H. E. F. Nielsen, J. Shaw, M. King, M.-A. Lea, N. Bax, “Antarctica just has this hero factor…”: Gendered barriers to Australian Antarctic research and remote fieldwork. PLOS ONE 14, e0209983 (2019). 30650104 10.1371/journal.pone.0209983 PMC6334902

[23] B. Carlin, T. Sikka, P. Hopkins, L. Braunholtz, L. Mair, Z. Pattison, Identifying the barriers to inclusion in field-based environmental sciences research. Stud. High. Educ. 49, 1652–1665 (2024).

[24] D. B. Sasse, Job-related mortality of wildlife workers in the United States, 1937-2000. Wildl. Soc. Bull. 31, 1000–1003 (2003).

[25] J. Linchant, J. Lisein, J. Semeki, P. Lejeune, C. Vermeulen, Are unmanned aircraft systems (UASs) the future of wildlife monitoring? A review of accomplishments and challenges. Mammal Rev. 45, 239–252 (2015).

[26] K. S. Christie, S. L. Gilbert, C. L. Brown, M. Hatfield, L. Hanson, Unmanned aircraft systems in wildlife research: Current and future applications of a transformative technology. Front. Ecol. Environ. 14, 241–251 (2016).

[27] M. D. Cantine, Dying to know: Death during geological fieldwork. Sediment. Rec. 19, 5–14 (2021).

[28] National Academies of Sciences, Engineering, and Medicine (NASEM), “Sexual harassment of women: Climate, culture, and consequences in academic sciences, engineering, and medicine” (The National Academies Press, 2018; https://nap.nationalacademies.org/catalog/24994/sexual-harassment-of-women-climate-culture-and-consequences-in-academic). 29894119

[29] K. Yarincik, A. Kelly, T. McGlynn, R. M. Verble, Best practices to promote field science safety. Integr. Comp. Biol. 63, 145–161 (2023). 37070952 10.1093/icb/icad014

[30] K. B. H. Clancy, R. G. Nelson, J. N. Rutherford, K. Hinde, Survey of Academic Field Experiences (SAFE): Trainees report harassment and assault. PLOS ONE 9, e102172 (2014). 25028932 10.1371/journal.pone.0102172 PMC4100871

[31] E. V. Fischer, B. Bloodhart, K. Rasmussen, I. B. Pollack, M. G. Hastings, E. Marin-Spiotta, A. R. Desai, J. P. Schwarz, S. Nesbitt, D. Hence, Leveraging field-campaign networks to identify sexual harassment in atmospheric science and pilot promising interventions. Bull. Am. Meteorol. Soc. 102, E2137–E2150 (2021).

[32] U.S. National Science Foundation Sexual Assault and Harassment Prevention and Response Program Office, “Report Based on Findings from the Sexual Assault and Harassment Climate Survey (SAHCS)” (OMB study 3145-0260, NSF, 2025); https://nsf-gov-resources.nsf.gov/files/USAP%20SAHCS%20Findings%20Report_Final_7.15.25.pdf.

[33] C. R. Perumalswami, A. K. Greene, K. A. Griffith, R. Jagsi, National Science Foundation grant awardees’ perspectives on Article X and sexual harassment in science. PLOS ONE 19, e0300762 (2024). 38687758 10.1371/journal.pone.0300762 PMC11060523

[34] M. St. Clair, “Sexual harassment in marine science” (Women in Ocean Science C.I.C., 2021; https://womeninoceanscience.com/sexual-harassment).

[35] S. E. Moss, M. Mahmoudi, STEM the bullying: An empirical investigation of abusive supervision in academic science. EClinicalMedicine 40, 101121 (2021). 34527894 10.1016/j.eclinm.2021.101121 PMC8433114

[36] A. Mattheis, E. Marín-Spiotta, S. Nandihalli, B. Schneider, R. T. Barnes, “Maybe this is just not the place for me:” Gender harassment and discrimination in the geosciences. PLOS ONE 17, e0268562 (2022). 35584104 10.1371/journal.pone.0268562 PMC9116675

[37] L. Mair, J. Hardwick, N. Mannion, L. Braunholtz, B. Carlin, T. Sikka, P. Hopkins, Z. Pattison, Improving university policies and risk assessment to support inclusive fieldwork in environmental sciences. Ecol. Solut. Evid. 6, e70109 (2025).

[38] F. Gill, C. Maclean, Knowing your place: Gender and reflexivity in two ethnographies. Sociol. Res. Online 7, 118–128 (2002).

[39] G. Sharp, E. Kremer, The safety dance: Confronting harassment, intimidation, and violence in the field. Sociol. Methodol. 36, 317–327 (2006).

[40] S. Giles, C. Jackson, N. Stephen, Barriers to fieldwork in undergraduate geoscience degrees. Nat. Rev. Earth Environ. 1, 77–78 (2020).

[41] J. Clarke, How much does hiking cost, really? (Advnture, 31 December 2022); https://advnture.com/features/hiking-costs.

[42] L. C. Loyola, Financial barriers to primatological field work: A brief commentary. Int. J. Primatol. 40, 465–467 (2019).

[43] L. E. Heath-Stout, E. M. Hannigan, Affording archaeology: How field school costs promote exclusivity. Adv. Archaeol. Pract. 8, 123–133 (2020).

[44] K. E. Davis, P. Meehan, C. Klehm, S. Kurncik, C. Cameron, Recommendations for safety education and training for graduate students directing field projects. Adv. Archaeol. Pract. 9, 74–80 (2021).

[45] L. D. Daniles, S. Lavallee, Better safe than sorry: Planning for safe and successful fieldwork. Bull. Ecol. Soc. Am. 95, 264–273 (2014).

[46] S. Z. Leidman, Å. K. Rennermalm, A. J. Broccoli, D. van As, M. R. van den Broeke, K. Steffen, A. Hubbard, Methods for predicting the likelihood of safe fieldwork conditions in harsh environments. Front. Earth Sci. 8, 10.3389/feart.2020.00260 (2020).

[47] D. Eisenberg, S. E. Gollust, E. Golberstein, J. Hefner, Prevalence and correlates of depression, anxiety, and suicidality among university students. Am. J. Orthopsychiatry 77, 534–542 (2007). 18194033 10.1037/0002-9432.77.4.534

[48] T. M. Evans, L. Bira, J. B. Gastelum, L. T. Weiss, N. L. Vanderford, Evidence for a mental health crisis in graduate education. Nat. Biotechnol. 36, 282–284 (2018). 29509732 10.1038/nbt.4089

[49] M. Cordaro, K. Howard, E. Schmiedehaus, S. Dailey, Faculty mental health and compassion fatigue: A call to the profession, a call to the institution. J. Work. Behav. Health 39, 318–346 (2024).

[50] L. A. Palinkas, F. Glogower, M. Dembert, K. Hansen, R. Smullen, Incidence of psychiatric disorders after extended residence in Antarctica. Int. J. Circumpolar Health 63, 157–168 (2004). 15253482 10.3402/ijch.v63i2.17702

[51] K. P. Eifling, How to prepare your team for an emergency—A preseason planning exercise in four parts. Adv. Archaeol. Pract. 9, 56–60 (2021).

[52] M. Gochfeld, C. D. Volz, J. Burger, S. Jewett, C. W. Powers, B. Friedlander, Developing a health and safety plan for hazardous field work in remote areas. J. Occup. Environ. Hyg. 3, 671–683 (2006). 17050349 10.1080/15459620601009201

[53] A. Ackerman, K. Yarincik, S. Murphy, I. Cetinić, A. Fundis, A. Miller, E. Shroyer, A. Busse, Q. Covington, A. DeSilva, A. Haupt, L. Johnson, C. Lee, L. Lorenzoni, B. Murphy, J. Ramarui, B. Rosenheim, D. Steinberg, Know before you go: A community-derived approach to planning for and preventing sexual harassment at oceanographic field sites. Oceanography 36, 38–43 (2023).

[54] A. E. Nordseth, J. R. Gerson, L. K. Aguilar, A. E. Dunham, A. Gentles, Z. Neale, E. Rebol, The Fieldwork Wellness Framework: A new approach to field research in ecology. Front. Ecol. Environ. 21, 297–303 (2023).

[55] D. Lewis, ‘Paralysed by anxiety’: Researchers speak about life in troubled ancient-DNA lab. Nature 572, 571–572 (2019). 31455916 10.1038/d41586-019-02540-5

[56] N. Whiting, T. Gunga, “Hostage takers demand ransom in exchange for release of Australian professor kidnapped in PNG,” *ABC News Australia*, 20 February 2023; https://abc.net.au/news/2023-02-21/png-hostage-takers-want-ransom-for-australian/102002566.

[57] K. Armstrong, “Papua New Guinea kidnap: Archaeologist Bryce Barker and colleagues freed,” *BBC News*, 26 February 2023; https://bbc.com/news/world-asia-64775769.

[58] Al Jazeera Staff, “South Africa’s Antarctica base hit by assault claims: What happened?” *Al Jazeera*, 21 March 2025; https://aljazeera.com/news/2025/3/21/south-africas-antarctica-base-hit-by-assault-claims-what-happened.

[59] A. Harwood, “Giulio Regeni’s last messages before his death in Egypt counter spy claims,” *The Guardian*, 14 June 2021; https://theguardian.com/global-development/2021/jun/14/giulio-regeni-last-messages-before-his-death-in-egypt-counter-spy-claims.

[60] N. Morales, K. B. O’Connell, S. McNulty, A. Berkowitz, G. Bowser, M. Giamellaro, M. N. Miriti, Promoting inclusion in ecological field experiences: Examining and overcoming barriers to a professional rite of passage. Bull. Ecol. Soc. Am. 101, e01742 (2020).

[61] E. N. Rudzki, S. E. Kuebbing, D. R. Clark, B. Gharaibeh, M. J. Janecka, R. Kramp, K. D. Kohl, T. Mastalski, M. E. B. Ohmer, M. M. Turcotte, C. L. Richards-Zawacki, A guide for developing a field research safety manual that explicitly considers risks for marginalized identities in the sciences. Methods Ecol. Evol. 13, 2318–2330 (2022).

[62] V. Ramírez-Castañeda, E. P. Westeen, J. Frederick, S. Amini, D. R. Wait, A. S. Achmadi, N. Andayani, E. Arida, U. Arifin, M. A. Bernal, E. Bonaccorso, M. B. Sanguila, R. M. Brown, J. Che, F. P. Condori, D. Hartiningtias, A. E. Hiller, D. T. Iskandar, R. A. Jiménez, R. Khelifa, R. Márquez, J. G. Martínez-Fonseca, J. L. Parra, J. V. Peñalba, L. Pinto-García, O. H. Razafindratsima, S. R. Ron, S. Souza, J. Supriatna, R. C. K. Bowie, C. Cicero, J. A. McGuire, R. D. Tarvin, A set of principles and practical suggestions for equitable fieldwork in biology. Proc. Natl. Acad. Sci. U.S.A. 119, e2122667119 (2022). 35972961 10.1073/pnas.2122667119 PMC9407469

[63] National Academies of Sciences, Engineering, and Medicine (NASEM), “Promising practices for addressing the underrepresentation of women in science, engineering, and medicine: Opening doors” (The National Academies Press, 2020; https://ncbi.nlm.nih.gov/sites/books/NBK554705/). 32134611

[64] K. J. Holland, A. E. Cipriano, T. Z. Huit, “The fear is palpable”: Service providers’ perceptions of mandatory reporting policies for sexual assault in higher education. Anal. Soc. Issues Public Policy 20, 66–89 (2019).

[65] B. Piexotto, C. Klehm, K. P. Eifling, Rethinking research sites as wilderness activity sites. Adv. Archaeol. Pract. 9, 1–9 (2021).

[66] CHIPS and Science Act, H.R. 4346, 117th Congress (2022). https://congress.gov/bill/117th-congress/house-bill/4346.

[67] K. P. Eifling, Mental health and the field research team. Adv. Archaeol. Pract. 9, 10–22 (2021).

[68] C. E. Colaninno, S. P. Lambert, E. L. Beahm, C. G. Drexler, Creating and supporting a harassment- and assault-free field school. Adv. Archaeol. Pract. 8, 111–122 (2020).

[69] M. R. Cronin, R. S. Beltran, E. S. Zavaleta, Beyond reporting: Proactive strategies for safer scientific fieldwork. Trends Ecol. Evol. 39, 213–216 (2024). 38320928 10.1016/j.tree.2024.01.003

[70] V. L. Banyard, Measurement and correlates of prosocial bystander behaviour: The case of interpersonal violence. Violence Vict. 23, 83–97 (2008). 18396583 10.1891/0886-6708.23.1.83

[71] V. L. Banyard, Who will help prevent sexual violence: Creating an ecological model of bystander intervention. Psychol. Violence 1, 216–229 (2011).

[72] M. R. Cronin, E. S. Zavaleta, R. S. Beltran, M. Esparza, A. R. Payne, V. Termini, J. Thompson, M. S. Jones, Testing the effectiveness of interactive training on sexual harassment and assault in field science. Sci. Rep. 14, 523 (2024). 38191560 10.1038/s41598-023-49203-0 PMC10774269

[73] A.-J. C. Demery, M. A. Pipkin, Safe fieldwork strategies for at-risk individuals, their supervisors and institutions. Nat. Ecol. Evol. 5, 5–9 (2021). 33046873 10.1038/s41559-020-01328-5

[74] J. J. Coon, N. B. Alexander, E. M. Smith, M. Spellman, I. M. Klimasmith, L. T. Allen-Custodio, T. E. Clarkberg, L. Lynch, D. Knutson, K. Fountain, M. Rivera, M. Scherz, L. K. Morrow, Best practices for LGBTQ+ inclusion during ecological fieldwork: Considering safety, cis/heteronormativity and structural barriers. J. Appl. Ecol. 60, 393–399 (2023).

[75] S. K. Flowers, K. O’Connell, V. M. McDermott, Crafting field station and marine lab communities for undergraduate diversity, equity, and inclusion. Bull. Ecol. Soc. Am. 102, e01908 (2021).

[76] A.-C. Devitz, Equipment repositories for accessibility: A model for improving access in field science. Integr. Comp. Biol. 63, 98–107 (2023). 37160341 10.1093/icb/icad024

